# Actionable Forecasting as a Determinant of Biological Adaptation

**DOI:** 10.1002/advs.202413153

**Published:** 2025-02-27

**Authors:** Jose M. G. Vilar, Leonor Saiz

**Affiliations:** ^1^ Biofisika Institute (CSIC UPV/EHU) and Department of Biochemistry and Molecular Biology University of the Basque Country UPV/EHU P.O. Box 644 Bilbao 48080 Spain; ^2^ IKERBASQUE Basque Foundation for Science Bilbao 48011 Spain; ^3^ Department of Biomedical Engineering University of California 451 E. Health Sciences Drive Davis CA 95616 USA

**Keywords:** circadian clocks, dynamic adaptation, fluctuations, neural networks, prediction

## Abstract

Organisms continuously adapt to changing environments to survive. Here, contrary to the prevailing view that predictive strategies are essential for perfect adaptation, it is shown that biological systems can precisely track their optimal state by adapting to a non‐anticipatory actionable target that integrates the current optimum with its rate of change. Predictive mechanisms, such as circadian rhythms, are beneficial for accurately inferring the actionable target when environmental sensing is slow or unreliable. A new mathematical framework is developed, showing that dynamics‐informed neural networks embodying these principles can efficiently capture biological adaptation even in noisy environments. These results provide fundamental insights into the interplay between forecasting, control, and inference in biological systems, redefining adaptation strategies and guiding the design of advanced adaptive biomolecular circuits.

## Graphical abstract

A new framework reveals how biological systems can achieve precise adaptation by tracking an actionable target that combines the current optimal state with its rate of change. This approach, implemented through dynamics‐informed neural networks, demonstrates that predictive mechanisms like circadian rhythms become beneficial when environmental sensing is unreliable, providing fundamental insights into biological adaptation strategies.

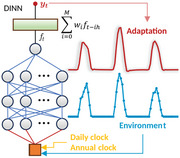

## 1 Introduction

In the search for general principles of living systems, extensive research has focused on network structure, information optimization, decision‐making, and evolution, among others.^[^ ^1^, ^2^, ^3^, ^4^, ^5^ ^]^ However, the role of forecasting capabilities of biological systems has not been as widely explored. Predicting the evolution of complex systems from continuously sensed real‐time data to control their behavior is a central challenge across biology and many other domains.^[^ ^6^, ^7^, ^8^, ^9^, ^10^, ^11^, ^12^, ^13^ ^]^ For example, advanced control strategies using machine learning have been applied to quantum systems, where Bayesian approaches and neural networks have been employed to optimize the control of inherently complex dynamics.^[^ ^12^, ^13^ ^]^ In biological systems, circadian rhythms exemplify inherently natural predictive behavior,^[^ ^14^ ^]^ yet how these mechanisms synergize with actionable strategies to mobilize resources remains an open question.^[^ ^15^, ^16^ ^]^

To address this gap, we develop a general framework to capture the effects of forecasting and actionability in the context of continuous adaptation. Understanding this connection is crucial because biological systems often adapt toward a potentially optimal state with a delay.^[^ ^17^, ^18^ ^]^ The longer the delay, the farther the system deviates from the optimal state. Whereas faster adaptation can reduce this deviation, it requires higher reaction rates and mobilization of resources, which is generally costly and detrimental.^[^ ^19^, ^20^ ^]^ Therefore, actionability requires a tradeoff between speed and resources. A central question, consequently, is how organisms can modify this tradeoff toward better adaptation with fewer resources.

We show that adapting toward a combination of the current environment and its rate of change, termed actionable target, rather than to the optimal state itself allows the system to precisely track a changing environment without delay. If the information about the current environment is precise and readily actionable, anticipatory mechanisms are not needed to implement perfect tracking, irrespective of the adaptation rate. However, predictability capabilities, such as those of circadian rhythms, can be advantageous when sensing the environment is unreliable or slow, as they help accurately infer the actionable target.

To explicitly study these tradeoffs, we focus on daily and seasonal clocks as well as on the current and recent environment as sources of information available for the adaptation processes. The main difference between these two types of sources is the instantaneous endogenous information provided by the clocks versus the exogenous environmental information that needs to be sensed and relayed through diverse signaling pathways, introducing delays before it becomes actionable. As an explicit quantitative environment, we consider solar radiation on the Earth's surface, which is directly relevant to the metabolism of photosynthetic organisms such as plants and cyanobacteria as well as to the behavior of virtually any system that is coupled to the environment, such as organisms and communities that follow the day‐night cycle.^[^ ^21^ ^]^

To implement the adaptation dynamics together with the forecasting approach, we developed a novel mathematical approach utilizing dynamics‐informed neural networks (DINNs).^[^ ^22^ ^]^ This approach allows us to test the performance of different actionable forecasting strategies coupled with the adaptation dynamics in noisy settings. By integrating these components, we provide new insights into the interplay between forecasting, control, and inference in biological systems, which not only redefines adaptation strategies but also sets the foundation for engineering advanced adaptive biomolecular circuits.

## 2 Results

### 2.1 Theory

The cellular system's state is generally determined by multiple variables, such as ATP levels, number of proteins, etc. We focus on the quantification of one of these variables. Explicitly, we consider the cellular state at time *t* described by *y_t_*. For a given environment at time *t*, there is an optimal cellular state $y_{t}^{o}$ that maximizes the growth rate. Near the optimal state, the growth rate is given by $r_{t}≃r_{t}^{o}-c_{t}\;{\left(y_{t}-y_{t}^{o}\right)}^{2}$, with *c_t_* ≡ −*d* ^2^ *r_t_*/(*dy_t_*)^2^, which is positive, and $r_{t}^{o}$ being the maximum growth rate. This dependence is important to define the optimization problem. Therefore, the objective is to minimize the mean square error (MSE) between the optimal and actual state over time, which is mathematically defined as $e_{MSE}=\lim_{T\to \infty }\frac{1}{T}\int_{0}^{T}{\left(y_{t}-y_{t}^{o}\right)}^{2}dt\;$. It is also useful to consider the root mean square error (RMSE) defined as $e_{RMSE}=\sqrt{e_{MSE}}\;$, which has the same units as the quantification of the cellular state.

We consider the dynamics given by

(1) $$\frac{dy_{t}}{dt}=b_{t}\left(f_{t}-y_{t}\right)$$

where *b_t_* is the adaptation rate of *y_t_* toward a function *f_t_* that depends on the environment. This equation can straightforwardly be solved as

(2) $$y_{t}=y_{0}e^{-\int_{0}^{t}b_{s}ds}+\int_{0}^{t}e^{-\int_{s}^{t}b_{z}dz}b_{s}f_{s}ds$$

Naïve adaptation would correspond to adaptation of the cellular state to the optimal value at a given time, i.e., $f_{t}=y_{t}^{o}\;$. In this scenario, *y_t_* will be different from $y_{t}^{o}$.

It is useful to write *f_t_* as $f_{t}-y_{t}^{o}+y_{t}^{o}$, substitute it in Equation 2, and integrate by parts the term $e^{-\int_{s}^{t}b_{z}dz}b_{s}y_{s}^{o}$, which makes use of the identity $\frac{d}{ds}e^{-\int_{s}^{t}b_{z}dz}y_{s}^{o}=e^{-\int_{s}^{t}b_{z}dz}b_{s}y_{s}^{o}+e^{-\int_{s}^{t}b_{z}dz}\frac{d}{ds}y_{s}^{o}$. The result

(3) $$y_{t}=y_{t}^{o}+\left(y_{0}-y_{0}^{o}\right)e^{-\int_{0}^{t}b_{s}ds}+\int_{0}^{t}e^{-\int_{s}^{t}b_{z}dz}b_{s}\left(f_{s}-y_{s}^{o}-\frac{1}{b_{s}}\frac{d}{ds}y_{s}^{o}\right)ds$$

shows that it is possible to perfectly track a changing optimal state, so that $y_{t}=y_{t}^{o}$ after the initial transient, if the system adapts toward

(4) $$f_{t}=y_{t}^{o}+\frac{1}{b_{t}}\frac{d}{dt}y_{t}^{o}$$

which we have termed the actionable target. Therefore, precise continuous tracking requires information on the optimal state, its changes, and the adaptation rate. The faster the adaptation rate, the smaller the dependence on the derivative. The explicit form of the actionable target shows that, in a constant environment, the system adapts toward the optimal state, as usually assumed. In general, however, precisely tracking the optimal state in variable environments requires adapting not to the optimal state but to a function that incorporates the changing rate, as dictated by the actionable target.

From a control theory point of view, the adaptation process is analogous to designing a controller that drives the system state to follow a desired reference trajectory, often referred to as asymptotic tracking.^[^ ^23^, ^24^ ^]^

Precise tracking does not in principle require information about the future or forecasting approaches. However, this property requires no delays in relaying the optimal state information. If there is a delay Δ*t* and the approach is applied straightforwardly, namely, $f_{t}=y_{t-\Delta t}^{o}+\frac{1}{b_{t}}\frac{d}{dt}y_{t-\Delta t}^{o}$, the system would track the delayed optimum value as $y_{t}=y_{t-\Delta t}^{o}\;$. Because $y_{t}^{o}-y_{t-\Delta t}^{o}≃\frac{d}{dt}y_{t}^{0}\Delta t$, the value of the MSE, $e_{MSE}≃\Delta t^{2}\left\langle {\left(\frac{d}{dt}y_{t}^{o}\right)}^{2}\right\rangle$, scales proportionally to the square of the delay and the average of the square of the rate of change of the optimal state.

In situations with delays and variable environments, adapting toward an estimate of the current actionable target could potentially be more efficient than adapting toward the actual delayed value. Accurately predicting the actionable target is crucial because the system would adapt toward $f_{t}={\hat{y}}_{t}^{o}+\frac{1}{b_{t}}\frac{d}{dt}{\hat{y}}_{t}^{o}$, where the hat indicates that the value ${\hat{y}}_{t}^{o}$ is a forecast of $y_{t}^{o}$ from past values.

### 2.2 Validation

**Figure 1:**
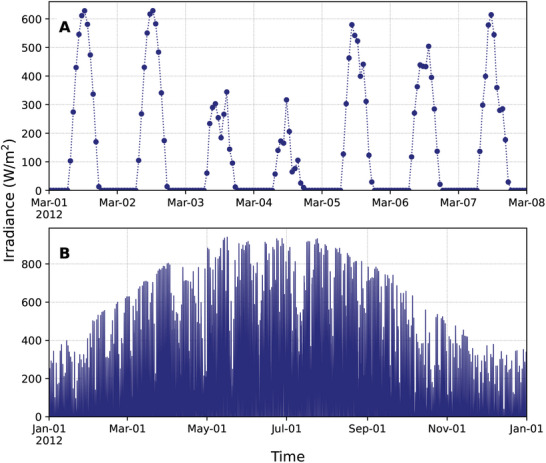
Temporal patterns of solar radiation on the Earth's surface at latitude 45° N and longitude 0° E illustrate the recurrent daily and annual fluctuations. A) Daily fluctuations shown over a one‐week period. B) Seasonal variations illustrated across a full year. Irradiance values were obtained from the PVGIS v5.2 database,^[^ ^25^ ^]^ developed and maintained by the European Commission Joint Research Centre (JRC). Data usage adheres to European Commission open data policies under the CC BY 4.0 license.

The theoretical framework developed in Section 2.1 establishes that perfect tracking of a changing optimal state requires adaptation toward an actionable target that combines both the current optimum and its rate of change. This mathematical insight has direct implications for biological systems that must continuously adapt to environmental fluctuations. To validate these theoretical predictions and demonstrate their practical relevance, we consider adaptation to the normalized hourly changes in solar radiation on the Earth's surface. As a normalization factor, we use the maximum radiation. As a representative location, we selected latitude 45° N and longitude 0° E. The specific latitude has marked seasonal effects superimposed on daily changes as well as weather patterns (**Figure** **1**). The longitude corresponds to the Greenwich meridian, for which the Coordinated Universal Time (UTC) corresponds to the mean solar time. The values, available in the PVGIS v5.2 database,^[^ ^25^ ^]^ quantify radiation at hourly resolution from the years 2005 to 2020 from satellite measurements. To validate the results, we consider data from the years 2015 to 2020. We keep the data from the years 2005 to 2014 for training in cases that rely on forecasts of the actionable target. This strategy ensures that the validation does not involve fitting.

**Figure 2:**
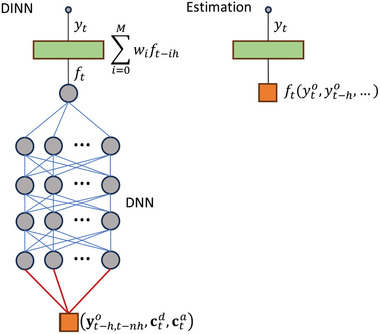
Implementation of continuous adaptation as a Dynamics‐Informed Neural Network (DINN). The panel on the left shows, from bottom to top, the structure of the DINN. The input, depicted as an orange square, consists of values of the vectors $y_{t-h,t-nh}^{o}$, $c_{t}^{d}$, and $c_{t}^{a}$, which connect to the input layer of the Deep Neural Network (DNN) (red lines). These vectors quantify the most recent *n* values of the optimal state before the time *t*, the phase of the daily clock, and the phase of the annual clock, respectively. The DNN consists of 4 dense layers, each containing 16 neurons with the Exponential Linear Unit (ELU) function activation. The output of the DNN feeds into a single‐neuron linear layer, which provides the actionable target *f_t_*. Blue lines connecting dense neurons (gray circles) represent trainable weights. The actionable target is the input of the convolutional layer (green rectangle) that implements the dynamics. The output of the convolutional layer is the state of the system *y_t_*. The panel on the right illustrates the approach with the numerical estimation of the actionable target. We considered the estimated value of *f_t_* (orange square) as the input of the convolutional layer.

The approach is embedded into a predictive framework through a dynamics‐informed neural network (**Figure** **2**). For the adaptation dynamics, we rely on the discretized integral representation (Equation 2) with a time‐independent adaptation rate constant (*b_t_* = *b*_0_ ) and without the transient term (long‐term behavior), which we implement as a convolutional layer of the neural network. The discretization follows the hourly sampling of solar radiation. Explicitly, we use $y_{t}=\sum_{i=0}^{M}w_{i}f_{t-ih\;}\;$ with the kernel *w_i_* given by $w_{0}=\frac{1}{2}\;b_{0}$ and $w_{i>0}=b_{0}\;e^{-b_{0}ih}$. Here, *M* is the size of the kernel and *h* represents a 1‐h interval. This result is obtained from the trapezoidal rule for numerical integration after a change of variables in the convolution of Equation 2. We use this approach with a single‐neuron linear layer with fixed weights to implement numerical approaches as well as with a trainable deep neural network (DNN) to capture potentially more complex predictive non‐linear relationships.

**Figure 3:**
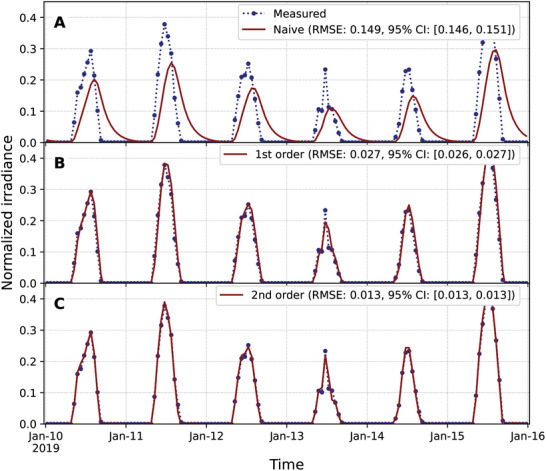
Adaptation toward the actionable target enables precise tracking even with discretely sampled data. The dotted blue line shows normalized measured values from the PVGIS v5.2 database.^[^ ^25^ ^]^ Solid red lines show the results for the adaptation toward A) the current optimal state, B) the current actionable target with a first‐order discrete backward approximation of the derivative, and C) the current actionable target with a second‐order discrete backward approximation of the derivative. The root mean square errors (RMSEs) between the measured values (dotted blue line) and tracked values (solid red lines) for the years 2015–2020 are shown in the legends of each panel, along with their 95% confidence intervals (CI) estimated using 24‐h block bootstrapping with 10000 iterations on 51840 hourly measurements.

The naïve adaptation mechanism, with $f_{t}=y_{t}^{o}\;$, leads to a substantial delay in tracking the optimal state (**Figure** **3**A), resulting in an RMSE of 0.149. Incorporating the optimal state rate of change through a first‐order approximation of the derivate, $f_{t}=y_{t}^{o}+\frac{1}{hb_{0}}\left(y_{t}^{o}-y_{t-h}^{o}\right),$ provides much better tracking than the naïve mechanism (Figure 3B) with an RMSE of 0.027. Trying to improve the estimation of the derivative through a centered second‐order approximation, $f_{t}=y_{t}^{o}+\frac{1}{2hb_{0}}\left(y_{t+h}^{o}-y_{t-h}^{o}\right)$, is not possible because it will require values from the future. Relying only on current and past values, a second‐order backward difference approximation of the derivative, $f_{t}=y_{t}^{o}+\frac{1}{2hb_{0}}\left(3y_{t}^{o}-4y_{t-h}^{o}+y_{t-2h}^{o}\right)$, further improves the tracking (Figure 3C) with an RMSE of 0.013. This result shows that if the actionable target is not delayed, there is accurate tracking without anticipatory mechanisms, even when the environment is noisy.

### 2.3 Delayed Inference and Extrapolation

**Figure 4:**
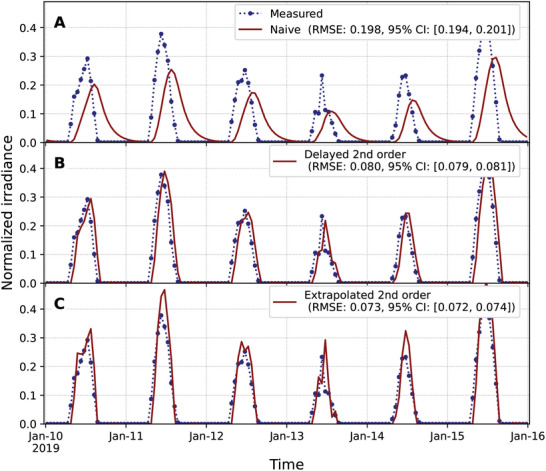
Delayed relay of the actionable target prevents perfect tracking but improves naïve adaptation. The dotted blue line shows normalized measured values from the PVGIS v5.2 database.^[^ ^25^ ^]^ Solid red lines show the results for the adaptation toward A) the 1‐h‐delayed optimal state, B) the 1‐h‐delayed actionable target, and C) the extrapolation to the current time from the 1‐h‐delayed actionable target. In both cases, the actionable target was computed with a second‐order discrete backward approximation of the derivative. The root mean square errors (RMSEs) between the measured values (dotted blue line) and tracked values (solid red lines) for the years 2015–2020 are shown in the legends of each panel, along with their 95% confidence intervals (CI) estimated using 24‐h block bootstrapping with 10000 iterations on 51840 hourly measurements.

The main limitation of this type of tracking lies in the ability to infer the value of the actionable target at time *t*. Explicitly, if only the past is actionable, namely, if there is a delay, the tracking ability is substantially reduced for the naïve case, $f_{t}=y_{t-h}^{o}\;$, with RMSE of 0.198 (**Figure** **4**A) and even for the second backward difference approximation of the derivative, $f_{t}=y_{t-h}^{o}+\frac{1}{2hb_{0}}\left(3y_{t-h}^{o}-4y_{t-2h}^{o}+y_{t-3h}^{o}\right)$, with RMSE of 0.080 (Figure 4B). Note that in the latter case, the RMSE is essentially the square root of the average value of ${\left(y_{t}^{o}-y_{t-h}^{o}\right)}^{2}$, which in this case is 0.082. We also considered the second‐order extrapolation of the optimal state and its derivative as ${\hat{y}}_{t}^{o}=y_{t-h}^{o}+\frac{1}{2}\left(3y_{t-h}^{o}-4y_{t-2h}^{o}+y_{t-3h}^{o}\right)$ and $\frac{d}{dt}\;{\hat{y}}_{t}^{o}=\frac{1}{2h}\;\left(5y_{t-h}^{o}-8y_{t-2h}^{o}+3y_{t-3h}^{o}\right)$, which leads to an RMSE of 0.073 (Figure 4C). Here, we have explicitly used $f_{t}={\hat{y}}_{t}^{o}+\frac{1}{b_{t}}\frac{d}{dt}{\hat{y}}_{t}^{o}$. In this case, linear extrapolation does not significantly improve the precision of the tracking because of the inherently noisy nature of the environment.

**Figure 5:**
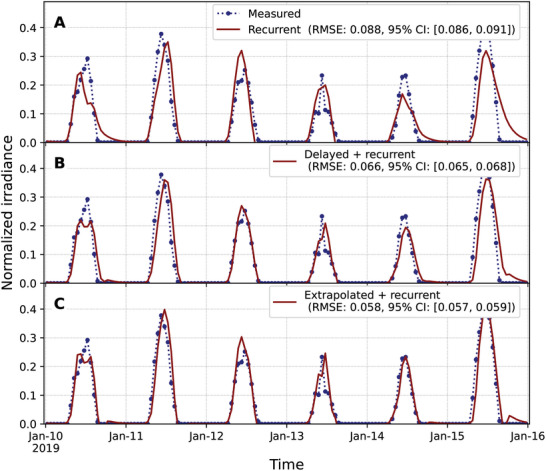
Estimation of the actionable target with long‐term recurrent changes improves tracking. The dotted blue line shows normalized measured values from the PVGIS v5.2 database.^[^ ^25^ ^]^ Solid red lines show the results for the adaptation toward A) the current time estimate of the actionable target with values of the previous day, B) the average of panel A and Figure 4B actionable targets, and C) the average of panel A and Figure 4C actionable targets. The root mean square errors (RMSEs) between the measured values (dotted blue line) and tracked values (solid red lines) for the years 2015–2020 are shown in the legends of each panel, along with their 95% confidence intervals (CI) estimated using 24‐h block bootstrapping with 10000 iterations on 51840 hourly measurements.

If the system has a recurrent component, such as those of daily cycles, it would be possible to estimate the optimal state and its change considering also values from one day earlier, namely, as ${\hat{y}}_{t}^{o}=y_{t-h}^{o}+y_{t-24h}^{o}-y_{t-24h-h}^{o}$ and $\frac{d}{dt}\;{\hat{y}}_{t}^{o}=\frac{1}{2h}\;\left(y_{t-24h+h}^{o}-y_{t-24h-h}^{o}\right)$. The resulting tracking has an RMSE of 0.088 (**Figure** **5**A). This type of approach, which can be improved to consider multiple days back in time, as in the Holt–Winters’ seasonal method,^[^ ^26^ ^]^ provides the system with periodic information. When the results of the delayed and the recurrent approaches are averaged together, the RMSE is reduced to 0.066 (Figure 5B), which is lower than the values obtained for each of them separately. The tracking error can be reduced further by considering the extrapolated instead of the delayed approach in the average with the recurrent information, which leads to an RMSE of 0.058. Such consistent error reductions upon averaging different estimations indicate that random fluctuations play a fundamental role in preventing the system from tracking the optimal value when forecasting is needed.

Overall, the results show that it is possible to substantially improve the naïve mechanism considering adaptation to the delayed actionable target, which can be further improved through forecasting mechanisms for the current optimal state and its time‐derivative contribution (Figures 4, 5). The forecasting mechanisms used so far should be considered as a baseline since they are linear, account for the recent past, and incorporate only the recurrences of 1 day before.

### 2.4 An Integrated Actionable Forecasting Strategy

Our analyses demonstrate the presence of both short‐ and long‐term information that can contribute to predicting changes in the optimal state. To generally address these dependencies, we consider a general Deep Neural Network (DNN) framework that explicitly incorporates short‐term, long‐term, and recurrent dependencies. The motivation for using a DNN is their ability to encode general functional dependencies between variables, their trainability properties, and their computational equivalence to biomolecular networks. Indeed, multiple results have shown explicitly that biomolecular networks, including enzymatic, signal transduction, and gene regulatory networks, can perform computations equivalent to those of artificial neural networks and other architectures in machine learning.^[^ ^27^, ^28^, ^29^, ^30^ ^]^

The potential role of recurrent effects enters the approach through a functional dependency on the time of the day (circadian) and the time of the year (circannual). Multiple organisms, from microorganisms as simple as bacteria to humans, have biomolecular mechanisms to respond to daily changes in environmental conditions. Circannual rhythms are needed to regulate physiological and behavioral processes over the changes that organisms experience through seasons, such as temperature and day‐length changes.^[^ ^31^, ^32^ ^]^ To account for these recurrent events, we consider explicitly two clocks that perform a circular motion in the unit circle in phase space as $c_{t}^{d}=\left(\sin\frac{2\pi t}{\tau_{D}},\cos\frac{2\pi t}{\tau_{D}}\right)$ for the daily changes and $c_{t}^{a}=\left(\sin\frac{2\pi t}{\tau_{Y}},\cos\frac{2\pi t}{\tau_{Y}}\right)$ for annual effects. Here, τ_*D*_ is the day length (24 h) and τ_*Y*_ is the year length (265.25 days). We do not delve into the potential biomolecular mechanisms for the clocks, which have been the subject of intense research.^[^ ^15^, ^33^, ^34^, ^35^, ^36^ ^]^ We only need their output as they are entrained by the environment.^[^ ^37^ ^]^ In this regard, clocks do not rely on precisely sensing the environment since only minimal coupling leads to perfect synchrony with external time. The recent environment is characterized through the *n*‐dimensional vector $y_{t-h,t-nh}^{o}=\left(y_{t-h}^{o},y_{t-2h}^{o}\cdot \cdot \cdot \;y_{t-nh}^{o}\right)$ with the most recent *n* values of the optimal state before the time *t*.

Explicitly, given $c_{t}^{d},$ $c_{t}^{a}$, and $y_{t-h,t-nh}^{o}$, we consider $f_{t}=f_{DNN}^{nda}\left(y_{t-h,t-nh},c_{t}^{d},c_{t}^{a}\right)$, $f_{t}=f_{DNN}^{nd}\left(y_{t-h,t-nh},c_{t}^{d}\right)$, and $f_{t}=f_{DNN}^{n}\left(y_{t-h,t-nh}\right)$ for different values of *n*. Here, $f_{DNN}^{n},$ $f_{DNN}^{nd},$ and $f_{DNN}^{nda}$ are the outputs of the DNNs for systems without, with circadian, and with both clocks, respectively. We analyze explicitly to what extent diverse types of systems can perfectly adapt to the changing optimal state depending on the information available and the network architecture implemented. We trained the networks with the data from the years 2005 to 2014. The validation was performed with data from the years 2015 to 2020 to test the predictive capabilities with unseen data.

**Figure 6:**
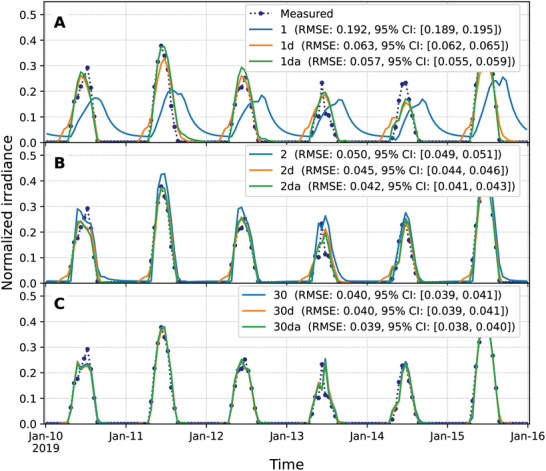
A dynamics‐informed neural network with daily and annual clocks reaches the limit of predictability with just two recent past values of the optimal state. The dotted blue line shows normalized measured values from the PVGIS v5.2 database.^[^ ^25^ ^]^ Solid lines show the results for the adaptation toward the estimate of the actionable target using a dynamics‐informed neural network (DINN) for systems without (blue), with daily (orange), and with daily and annual (green) clocks for A) 1, B) 2, and C) 30 actionable past values of the optimal state. The root mean square errors (RMSEs) between the measured values (dotted blue line) and tracked values (solid lines) for the years 2015–2020 are shown in the legends of each panel, along with their 95% confidence intervals (CI) estimated using 24‐h block bootstrapping with 10000 iterations on 51840 hourly measurements.

For systems that can only act on information about the most recent past value of the optimal state, *n* = 1, the presence of clocks significantly increases the ability to track the optimal state (**Figure** **6**A). The presence of clocks brings the tracking capabilities of the DNNs along the lines of the combined extrapolated and recurrent approaches (Figure 5C). In the absence of clocks, in contrast, the capabilities of a DNN do not substantially improve the results of naïve adaptation to the last optimal value available (Figure 4A). For systems that can act on information about the two most recent past values of the optimal state, *n* = 2, the differences between approaches are not as marked (Figure 6B). In all the cases, the DNN outperforms the results from the linear estimates of the actionable target (Figures 4, 5). For large numbers of actionable past values, such as *n* = 30, the results are essentially the same for all the DNNs, with an RMSE of about 0.040 (Figure 6C).

**Figure 7:**
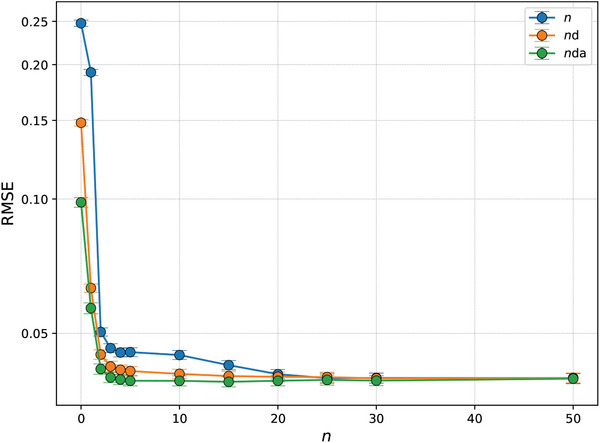
Dynamics‐informed neural network approaches without, with daily, and with daily and annual clocks become essentially equally accurate at *n* ≃ 25 as the number of actionable past values of the environment increases. The root mean square errors (RMSEs) for the years 2015–2020 between the measured and tracked values is shown as a function of the number of past actionable values, denoted by *n*, for systems without (blue), with daily (orange), and with daily and annual (green) clocks. To better visualize variations across a wide range of RMSE values, the *y*‐axis uses a logarithmic scale. Error bars represent the 95% confidence intervals for the RMSE, estimated using 24‐h block bootstrapping with 10000 iterations on 51840 hourly measurements.

An exhaustive analysis for values up to *n* = 50 shows that all the approaches become essentially equally accurate at *n* ≃ 25 as the number of actionable values increases (**Figure** **7**). The most salient result is the ability of systems with daily and annual clocks to reach nearly maximum tracking accuracy, as exemplified in Figure 6B, by just acting on two past values of the optimal state at *n* = 2. In this case, information about the recurrent environment is encoded as a function of the time of the day and the day of the year through the training of the network along the environment history.

This efficiency gain has profound implications for biological systems, where maintaining and processing large amounts of historical data would impose significant metabolic and regulatory costs. The ability to achieve optimal adaptation with minimal historical data represents a remarkable example of how evolutionary processes have optimized information processing in biological systems. This result also has practical implications for the design of adaptive biomolecular circuits. It suggests that incorporating clock‐like mechanisms could significantly reduce the complexity and resource requirements of synthetic biological systems while maintaining high‐performance levels.

## 3 Discussion

Continuously adapting to the optimal state in a changing environment is essential for the survival of virtually all organisms. Multiple biomolecular processes sense the environment and respond to current conditions to adapt to them. These processes include, among others, transcription, translation, phosphorylation, methylation, and changing multiple intracellular molecular concentrations.^[^ ^38^, ^39^ ^]^ This reactive approach implies a delay in reaching the optimal state, determined by the speed of the cellular process.^[^ ^40^, ^41^, ^42^ ^]^ Besides reactive approaches, there are also proactive approaches, such as the regulatory, metabolic, and physiological oscillations that match daily and annual changes.^[^ ^14^ ^]^ In many cases, these oscillations persist even in organisms artificially kept under constant environments. This persistence has been attributed to the need for organisms to anticipate changes in the natural environment.^[^ ^37^ ^]^

Here, we have shown that it is possible to continuously track the optimal state as it changes by adapting toward a combination of the current optimal state and its rate of change, termed the actionable target. This quantity, and hence accurate adaptation to a changing environment, does not depend on the future values of the environment. The fundamental limitation for accurate temporal tracking is obtaining an actionable target accurately. In general, inherent delays would prevent obtaining current values, and intrinsic fluctuations would prevent obtaining precise values.^[^ ^43^ ^]^ Therefore, the limitations for continuous adaptation are not anticipating the future but obtaining a reliable quantification of the present.

In addition to the optimal state of the system, a fundamental quantity is its rate of change. Remarkably, change sensing is widely present across organisms. The most studied example is bacterial chemotaxis, which relies on sensing temporal changes in nutrient concentrations as the bacterium moves through an inhomogeneous nutrient distribution.^[^ ^44^ ^]^ In higher organisms, there are multiple examples of complex pathways for sensing concentrations and their changes that can even perform complex computations.^[^ ^45^, ^46^ ^]^ However, the relay of this information typically follows substantial delays.

In this context, our results show that biological clocks are essential to provide a reliable time to be used with the underlying networks to estimate the expected current changes that mimic historical changes at a given time of the day. As the rate of change is encoded through the phase of the clocks, it does not depend on sensing the environment. Our results demonstrate that, in a variable, uncertain environment, biological clocks combined with sensing recent past values of the optimal state further increase the reliability of the estimation of the actionable target. In the case of tracking the solar radiation on the Earth's surface, we have shown that only two recent values are needed to reach the limits of precise tracking with circadian and circannual clocks. The need to sense only two values is equivalent to sensing the environment and its rate of change, which are widely present capabilities of organisms. When clocks are absent, the limit plateaus at about 25 values. Due to the inherent constraints of sensing, it is unclear how a bimolecular system would record, act upon, and process such an amount of temporal information, especially as information becomes prohibitively costly as its demand increases.^[^ ^47^ ^]^ In contrast, actionable forecasting can be implemented efficiently through biological functions that rely on oscillatory behavior.

Beyond its implications for understanding biological adaptation, our results are also relevant in fields that require real‐time adaptation to dynamic conditions, such as robotics, autonomous systems, and artificial intelligence.^[^ ^6^, ^7^, ^8^, ^9^, ^10^, ^11^ ^]^ Notably, in complex scenarios like quantum control, recent advances have demonstrated the utility of Bayesian machine learning and neural networks to optimize control strategies and solve inverse problems in high‐dimensional spaces.^[^ ^12^, ^13^ ^]^ These studies reveal parallels between controlling quantum systems and adapting biological systems to changing environments. However, while quantum systems primarily benefit from probabilistic control frameworks, biological systems must rely on actionable targets derived from real‐time and historical data. The dynamics‐informed neural network (DINN) framework, which integrates data‐driven learning with underlying dynamical models, offers the potential to develop more robust and data‐efficient control strategies in these contexts. In particular, actionable forecasting can guide the design of adaptive control policies that operate effectively in complex, time‐varying environments, where precise predictions are challenging but continuous tracking is essential.

By demonstrating how actionable forecasting can be implemented through a biologically plausible learning architecture, our work paves the way for biomimetic approaches to adaptive control and decision‐making. This approach can broadly extend to multiple applications, from optimizing the behavior of autonomous systems to improving the efficiency of artificial intelligence algorithms in uncertain environments.

Therefore, our results provide new insights into fundamental principles and constraints of biological adaptation, emphasizing the importance of accurately tracking the present environment instead of relying on future predictions. Through the concept of the actionable target, we illustrate how organisms can achieve near‐optimal adaptation in changing environments, efficiently implemented using dynamics‐informed neural networks. Through the integration of key concepts from biology, control theory, and machine learning, our results reveal the foundation for a unified understanding of dynamic adaptation across natural and artificial systems.

## 4 Experimental Section

### Field Data

The data was retrieved from the PVGIS v5.2 database^[^ ^25^ ^]^ using the web API command “https://re.jrc.ec.europa.eu/api/v5_2/seriescalc?lat=45&lon=0&raddatabase=PVGIS‐ERA5.” Data from the years 2015 to 2020 was used for the approach validation with numerical estimates of the actionable target and performance assessment with the DINN implementation. The data from the years 2005 to 2014 was used for training the DNN weights. The PVGIS v5.2 database is developed and maintained by the European Commission Joint Research Centre (JRC). The field data is provided under the European Commission's open data policies.

### Convolutional Layer Kernel Parametrization

The integral representation of the dynamics through Equation 2 was considered with a time‐independent adaptation rate constant and without the transient, which after a change of variable in the convolution leads to $y_{t}=\int_{0}^{t}e^{-b_{0}\left(t-s\right)}b_{0}f_{s}ds=\int_{0}^{t}e^{-b_{0}s}b_{0}f_{t-s}ds\;$. Using the trapezoidal rule, the integral is approximated as $y_{t}≃b_{0}\sum_{i=0}^{M}\frac{1}{2}\left(e^{-b_{0}ih}f_{t-ih\;}+e^{-b_{0}\left(i+1\right)h}f_{t-(i+1)h}\right)≃\sum_{i=0}^{M}w_{i}f_{t-ih\;}$, which defines the kernel *w_i_*. $b_{0}=\frac{1}{3h}$ was selected as a typical value, and *M* = 25 so that $\frac{1}{2}\;e^{-b_{0}\left(M+1\right)h}=8.6\times 10^{-5}$ and subsequent terms for longer delays were negligible. This value of *M* corresponds to a kernel size of 24 h.

### Computational Implementation

The overall approach was implemented in Keras^[^ ^48^ ^]^ and TensorFlow.^[^ ^49^ ^]^

For the DINN, a DNN with 4 dense layers, each containing 16 neurons with the Exponential Linear Unit (ELU) function activation was considered. As input of the DNN, values of the vectors $y_{t-h,t-nh}^{o}$, $c_{t}^{d},$ and $c_{t}^{a}$ were used. The output of the DNN was combined into a single‐neuron linear layer, which provided the actionable target *f_t_* used as the input of the convolutional layer that implements the dynamics. The MSE between the output of the convolutional layer, *y_t_*, and the optimal state, defined as the normalized solar irradiance $y_{t}^{o}$, was used as a loss function. Training of the overall network was performed over 365 intervals, 744‐h long each, as a single batch. The potentially overlapping intervals were chosen randomly from the years 2005 to 2014. Validation was performed over 72 consecutive intervals, 744‐h long each and spaced by 30 days, covering the years 2015 to 2020.

For the numerical estimation of the actionable target, the estimated value of *f_t_* was considered as the input of the convolutional layer. No training was involved. Validation was performed exactly as for the DINN.

### Statistical Analysis

Data preprocessing involved normalizing the solar radiation values from the PVGIS v5.2 database by dividing by the maximum radiation value for the entire dataset (2005–2020). All normalized values fall within the range [0,1]. Training of the neural networks utilized data from 2005–2014, consisting of 365 intervals of 744 h each (271560 total nonunique hourly measurements). For validation analyses, hourly data from 2015–2020, comprising 72 consecutive intervals of 744 h each was used. Given the kernel size of 24 h, each 744‐h interval resulted in 720 values of $y_{t}^{o}$, corresponding to 51840 total unique hourly values.

Performance evaluation utilized the Root Mean Square Error (RMSE) metric, calculated as $e_{RMSE}=\sqrt{e_{MSE}}\;$, where *e_MSE_* represents the mean square error between the optimal and actual state over time. The RMSE maintains the same units as the normalized solar radiation measurements, enabling direct interpretation of the error magnitude. Statistical significance in model performance was evaluated using 95% confidence intervals (CI) for the RMSE values. The CIs were estimated through block bootstrapping with a 24‐h block size and 10000 iterations. The full validation dataset of 51840 hourly measurements was reshaped into 2160 blocks of 24 h each. These blocks were randomly sampled with replacements to create 10000 bootstrap samples. The 2.5th and 97.5th percentiles of the resulting RMSE distribution were used as the lower and upper bounds of the 95% CI. No post‐hoc tests were required as the analysis focused on direct comparisons of RMSE values with their respective confidence intervals.

For the dynamics‐informed neural network training, the Adamax optimizer with a dynamic learning rate schedule was employed. The training was performed in batches containing all samples, with each training cycle consisting of 800 epochs. The training process included multiple optimization rounds with decreasing learning rates until convergence, defined as less than 10^−5^ change in validation loss between successive rounds. The computational analyses were performed using Python 3.11 with TensorFlow 2.15 and Keras 2.15 frameworks in a CUDA‐enabled environment (CUDA 12.4).

## Conflict of Interest

The authors declare no conflict of interest.

## Data Availability

Data sharing is not applicable to this article as no new data were created or analyzed in this study.

## References

[1] T. Yamada , P. Bork , Nat. Rev. Mol. Cell Biol. 2009, 10, 791. 19851337 10.1038/nrm2787

[2] L. H. Hartwell , J. J. Hopfield , S. Leibler , A. W. Murray , Nature 1999, 402, C47. 10591225 10.1038/35011540

[3] E. Kussell , S. Leibler , Science 2005, 309, 2075. 16123265 10.1126/science.1114383

[4] G. Balazsi , A. van Oudenaarden , J. J. Collins , Cell 2011, 144, 910. 21414483 10.1016/j.cell.2011.01.030 PMC3068611

[5] R. M. May , Trends in Ecology & Evolution 2006, 21, 394. 16815438 10.1016/j.tree.2006.03.013

[6] X. Wu , X. Zhu , G.‐Q. Wu , W. Ding , IEEE transactions on knowledge data engineering 2014, 26, 2382. 10.1109/TKDE.2014.2300480 PMC4199244 25328361

[7] M. Schwenzer , M. Ay , T. Bergs , D. Abel , The International Journal of Advanced Manufacturing Technology 2021, 117, 1327.

[8] J. M. G. Vilar , arXiv:1902.04337 2019.

[9] J.‐B. Lugagne , C. M. Blassick , M. J. Dunlop , Nat. Commun. 2024, 15, 2148. 38459057 10.1038/s41467-024-46361-1 PMC10923782

[10] R. S. Parker , F. J. Doyle , N. A. Peppas , IEEE Trans. Biomed. Eng. 1999, 46, 148. 9932336 10.1109/10.740877

[11] M. Anvari , E. Proedrou , B. Schäfer , C. Beck , H. Kantz , M. Timme , Nat. Commun. 2022, 13, 4593. 35933555 10.1038/s41467-022-31942-9 PMC9357012

[12] X. Wang , A. Kumar , C. R. Shelton , B. M. Wong , Phys. Chem. Chem. Phys. 2020, 22, 22889. 32935687 10.1039/d0cp03694c

[13] M. F. Lazin , C. R. Shelton , S. N. Sandhofer , B. M. Wong , Machine Learning: Science and Technology 2023, 4, 045014.

[14] A. T. Winfree , The Geometry of Biological Time, Springer, New York, NY, 2001.

[15] A. Patke , M. W. Young , S. Axelrod , Nat. Rev. Mol. Cell Biol. 2020, 21, 67. 31768006 10.1038/s41580-019-0179-2

[16] Y. Ouyang , C. R. Andersson , T. Kondo , S. S. Golden , C. H. Johnson , Proc. Natl. Acad. Sci. USA 1998, 95, 8660. 9671734 10.1073/pnas.95.15.8660 PMC21132

[17] G. Lambert , E. Kussell , PLoS Genet. 2014, 10, e1004556. 25255314 10.1371/journal.pgen.1004556 PMC4177670

[18] J. M. G. Vilar , R. Jansen , C. Sander , PLoS Comput. Biol. 2006, 2, e3. 16446785 10.1371/journal.pcbi.0020003 PMC1356091

[19] P. Salvy , V. Hatzimanikatis , Proc. Natl. Acad. Sci. USA 2021, 118, e2013836118. 33602812 10.1073/pnas.2013836118 PMC7923608

[20] G. Lan , P. Sartori , S. Neumann , V. Sourjik , Y. Tu , Nat. Phys. 2012, 8, 422. 22737175 10.1038/nphys2276 PMC3378065

[21] C. R. McClung , The Plant Cell 2006, 18, 792. 16595397 10.1105/tpc.106.040980 PMC1425852

[22] J. M. G. Vilar , L. Saiz , Sci. Adv. 2023, 9, eadf0673. 37450598 10.1126/sciadv.adf0673 PMC10348669

[23] H. K. Khalil , Nonlinear systems, Prentice Hall, Upper Saddle River, N.J. 2002.

[24] K. J. Åström , B. r. Wittenmark , Adaptive control, Dover Publications, Mineola, N.Y. 2008.

[25] T. Huld , R. Müller , A. Gambardella , Sol. Energy 2012, 86, 1803.

[26] P. R. Winters , Management science 1960, 6, 324.

[27] S. Okumura , G. Gines , N. Lobato‐Dauzier , A. Baccouche , R. Deteix , T. Fujii , Y. Rondelez , A. J. Genot , Nature 2022, 610, 496. 36261553 10.1038/s41586-022-05218-7

[28] J. Kim , J. Hopfield , E. Winfree , Advances in neural information processing systems 2004, 17, 681.

[29] K. M. Cherry , L. Qian , Nature 2018, 559, 370. 29973727 10.1038/s41586-018-0289-6

[30] C. G. Evans , J. O'Brien , E. Winfree , A. Murugan , Nature 2024, 625, 500. 38233621 10.1038/s41586-023-06890-z PMC10794147

[31] G. Lincoln , D. Hazlerigg , Society of Reproduction and Fertility supplement 2019, 67, 171. 21755671

[32] M. E. Visser , S. P. Caro , K. Van Oers , S. V. Schaper , B. Helm , Philosophical Transactions of the Royal Society B: Biological Sciences 2010, 365, 3113. 10.1098/rstb.2010.0111 PMC2981940 20819807

[33] J. M. G. Vilar , H. Y. Kueh , N. Barkai , S. Leibler , Proc Natl Acad Sci. U S A 2002, 99, 5988. 11972055 10.1073/pnas.092133899 PMC122889

[34] W. Pittayakanchit , Z. Lu , J. Chew , M. J. Rust , A. Murugan , Elife 2018, 7, e37624. 29988019 10.7554/eLife.37624 PMC6059770

[35] P. Meyer , L. Saez , M. W. Young , Science 2006, 311, 226. 16410523 10.1126/science.1118126

[36] M. Monti , D. K. Lubensky , P. R. Ten Wolde , Phys. Rev. Lett. 2018, 121, 078101. 30169070 10.1103/PhysRevLett.121.078101

[37] D. A. Golombek , R. E. Rosenstein , Physiol. Rev. 2010, 90, 1063. 20664079 10.1152/physrev.00009.2009

[38] W. Kolch , M. Halasz , M. Granovskaya , B. N. Kholodenko , Nat. Rev. Cancer 2015, 15, 515. 26289315 10.1038/nrc3983

[39] F. Jacob , J. Monod , J. Mol. Biol. 1961, 3, 318. 13718526 10.1016/s0022-2836(61)80072-7

[40] J. Monod , Annu. Rev. Microbiol. 1949, 3, 371.

[41] J. M. G. Vilar , L. Saiz , Biophys. J. 2013, 104, 2574. 23790365 10.1016/j.bpj.2013.04.032 PMC3686343

[42] J. M. G. Vilar , L. Saiz , Cell Syst 2024, 15, 639. 38981487 10.1016/j.cels.2024.06.002

[43] L. Saiz , J. M. G. Vilar , Mol Syst Biol 2006, 2, 0024. 10.1038/msb4100061 PMC1681493 16738569

[44] T. S. Shimizu , Y. Tu , H. C. Berg , Mol Syst Biol 2010, 6, 382. 20571531 10.1038/msb.2010.37 PMC2913400

[45] Y. E. Antebi , J. M. Linton , H. Klumpe , B. Bintu , M. Gong , C. Su , R. McCardell , M. B. Elowitz , CellCell 2017, 170, 1184. 10.1016/j.cell.2017.08.015 PMC5612783 28886385

[46] J. M. G. Vilar , L. Saiz , Cell Syst 2017, 5, 316. 29073371 10.1016/j.cels.2017.10.006

[47] A. J. Tjalma , V. Galstyan , J. Goedhart , L. Slim , N. B. Becker , P. R. ten Wolde , Proc. Natl. Acad. Sci. USA 2023, 120, e2303078120. 37792515 10.1073/pnas.2303078120 PMC10576116

[48] F. Chollet , Deep learning with Python, Manning Publications Co., Shelter Island, NY 2018.

[49] M. Abadi , P. Barham , J. Chen , Z. Chen , A. Davis , J. Dean , M. Devin , S. Ghemawat , G. Irving , M. Isard , 12th USENIX symposium on operating systems design and implementation (OSDI 16) 2016, 265.

